# Supplementary material for: Antioxidative Role of Hygrophila erecta (Brum. F.) Hochr. on UV-Induced Photoaging of Dermal Fibroblasts and Melanoma Cells
Source: Antioxidants (Basel). 2022 Jul 2;11(7):1317. doi: 10.3390/antiox11071317 (PMC9311957; doi:10.3390/antiox11071317)
Supplement: Supplementary file 1 [file antioxidants-11-01317-s001.zip › antioxidants-1784222-supplementary.pdf]

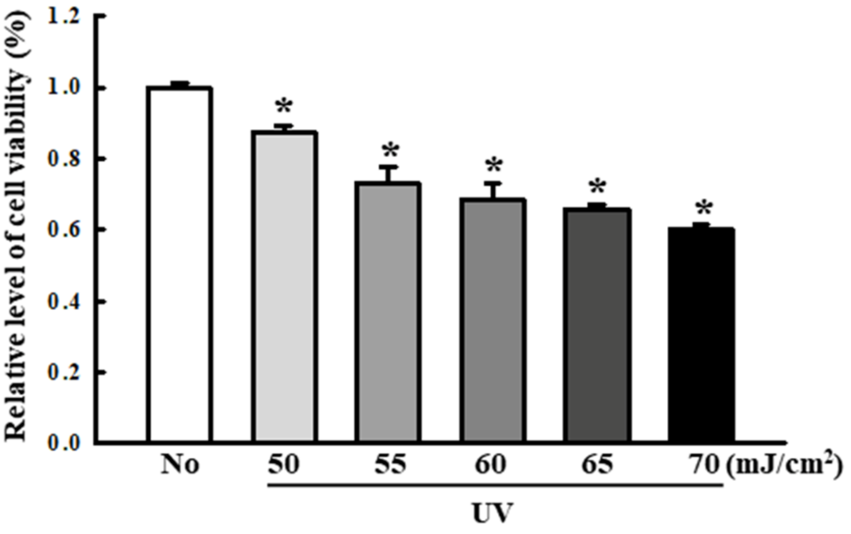

Figure S1. Determination of the optimal UV dosage. Three wells per group were used in the MTT assay, and the optical density was measured in duplicate. Data are reported as the means ± SD. *, $p<0.05$ relative to the No treated group. Abbreviations: UV, Ultraviolet; MTT, 3-(4,5-Dimethylthiazol-2-yl)-2,5-Diphenyltetrazolium Bromide.

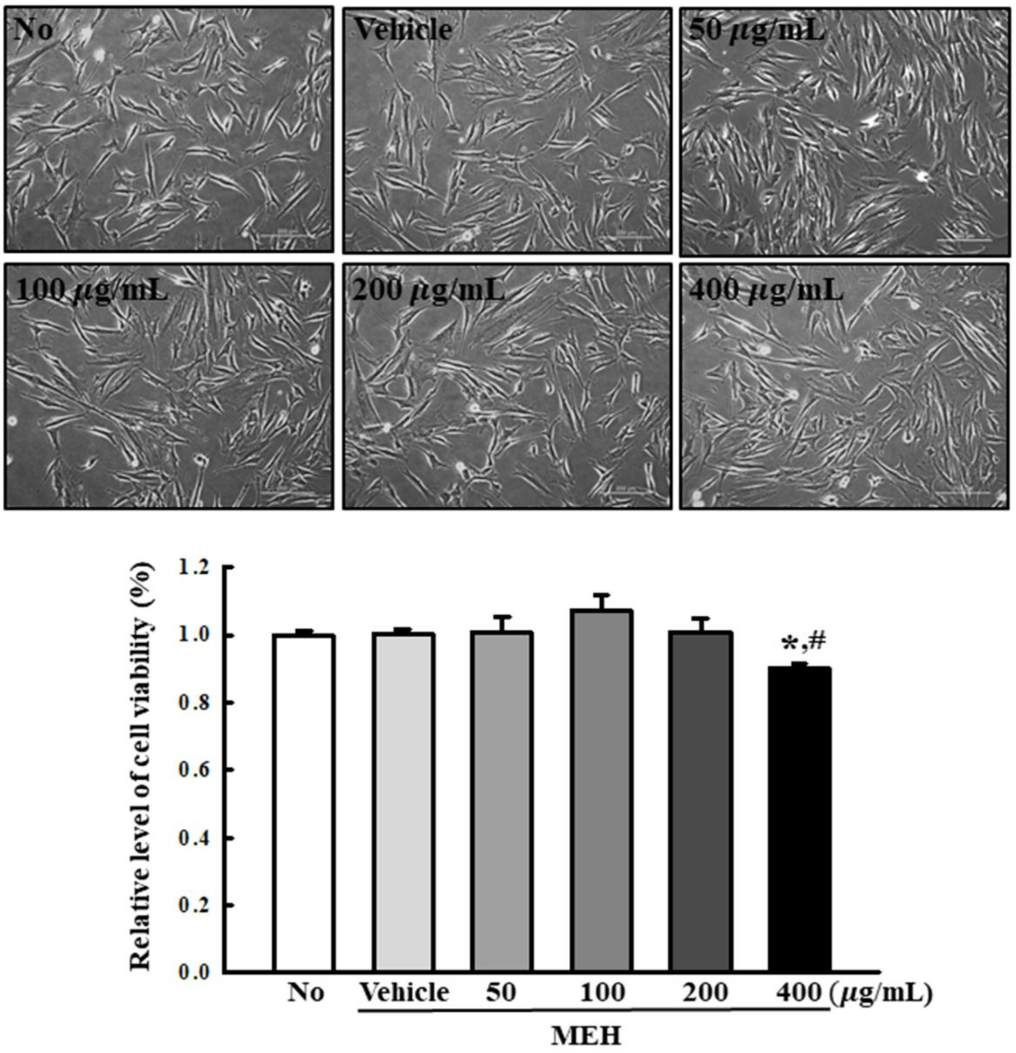

Figure S2. Determination of the optimal MEH dosage in NHDF cells. Three wells per group were used in the MTT assay, and the optical density was measured in triplicate. The data are reported as the means ± SD. Abbreviations: MEH, Methanol extracts of Hygrophila erecta (Brum. F.) Hochr; MTT, 3-(4,5-Dimethylthiazol-2-yl)-2,5-Diphenyltetrazolium Bromide.

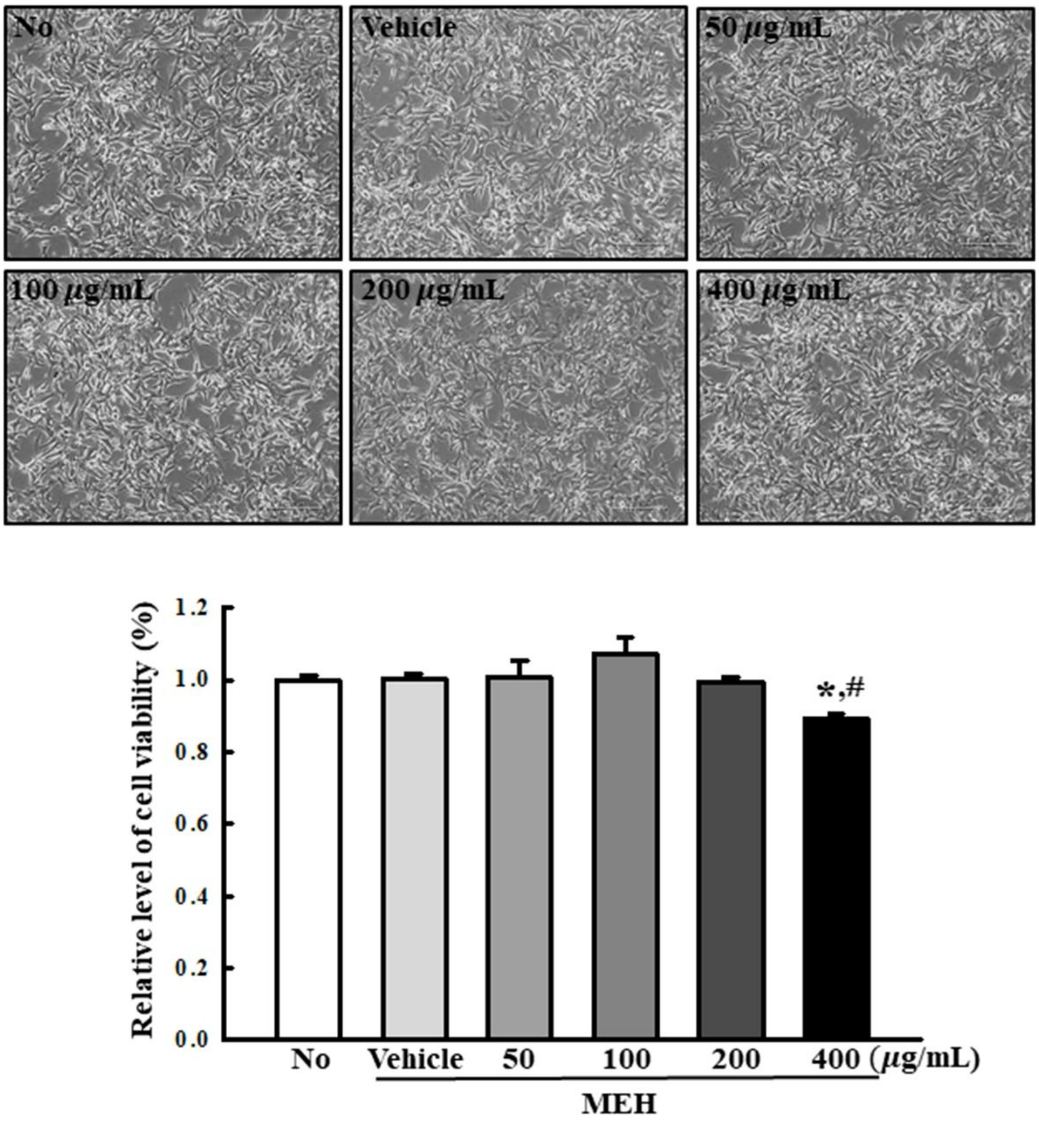

Figure S3. Determination of the optimal MEH dosage in B16F1 cells. Three wells per group were used in the MTT assay, and the optical density was measured in triplicate. The data are reported as the means ± SD. Abbreviations: MEH, Methanol extracts of Hygrophila erecta (Brum. F.) Hochr; MTT, 3-(4,5-Dimethylthiazol-2-yl)-2,5-Diphenyltetrazolium Bromide.
